# Body Appreciation, Depressive Symptoms, and Self-Esteem in Pregnant and Postpartum Brazilian Women

**DOI:** 10.3389/fgwh.2022.834040

**Published:** 2022-03-17

**Authors:** Juliana Fernandes Filgueiras Meireles, Clara Mockdece Neves, Ana Carolina Soares Amaral, Fabiane Frota da Rocha Morgado, Maria Elisa Caputo Ferreira

**Affiliations:** ^1^Laboratory of Body Studies, Physical Education and Sports Faculty, Federal University of Juiz de Fora, Juiz de Fora, Brazil; ^2^Federal Institute of Education, Science and Technology of Southeast of Minas Gerais, Barbacena, Brazil; ^3^Department of Physical Education and Sports, Institute of Education, Rural Federal University of Rio de Janeiro, Rio de Janeiro, Brazil

**Keywords:** body image, pregnancy, postpartum period, mental health, maternal welfare, cross-sectional study

## Abstract

**Background:**

During pregnancy and the postpartum period, women experience tremendous biopsychosocial changes in a short period of time. Poor body appreciation, depressive symptoms, and low self-esteem during the perinatal period may cause negative consequences for both the mother and the infant's physiological and psychological health. The aim of this study was to analyze the differences in body appreciation, depressive symptoms, and self-esteem between the three gestational trimesters and the postpartum period.

**Methods:**

Participants (*N* = 1,423 pregnant and postpartum Brazilian women), aged between 18 and 42 years old (*M* = 29.22; SD = ±5.72), answered questionnaires on body appreciation, depressive symptoms, and self-esteem. BMI was self-reported. Descriptive and nonparametric covariance analysis were performed, comparing women who were in the first, second, and third trimesters and the postpartum period.

**Results:**

Body appreciation was significantly higher among women in the third trimester compared to those in the first and second trimester. However, it was lower for women in all three gestational trimesters than for those in the postpartum. There was no difference in self-esteem during pregnancy, but it was significantly lower in the postpartum group. Similarly, depressive symptoms did not vary through pregnant groups but it was significantly higher in the postpartum group.

**Conclusions:**

The results showed that the postpartum period can be a difficult experience for women. They presented poor body appreciation and lower self-esteem and higher depressive symptoms compared to the pregnancy period. Therefore, it is necessary for public health policies to support women in this period, preserving their mental health and making this experience more positive.

## Introduction

During pregnancy and the postpartum period, women experience several biopsychosocial changes in a short period of time (1–4). The gestational period is divided into first, second, and third trimesters. In the first trimester (weeks 1–12), the woman begins to gain weight, and mood swings may occur, such as moments of depression and agitation (5). The second trimester (weeks 13–25) is characterized by the onset of fetal movements and the belly becomes more visible (5). This trimester is also considered the most emotionally stable (6). In the third trimester (from week 26 onwards), there is a greater increase in maternal weight (5, 7) and this is the period of greatest apprehension for most women due to proximity to delivery (6). Up to 6 months after delivery, women deal with the complex recovery of the body from changes that occurred during pregnancy and childbirth (5), and they are susceptible to psychological vulnerability (3, 8).

Mental health disorders affect many women during pregnancy and the postpartum period (3, 8, 9). Low self-esteem, depressive symptoms, and low levels of body appreciation during pregnancy and postpartum can have negative consequences for the well-being of the woman, her partner, and her family, and for the birth and development of the child (10). In particular, ~30–70% of women who have given birth have had lower self-esteem after delivery (11). Low self-esteem causes a series of social and psychological problems, such as degraded marital relationships, depression, bulimia, lower mother-fetus attachment, and early termination of breastfeeding (1, 11, 12). Associated with this low self-esteem, about 20% of women may develop depression during pregnancy (13), and in the postpartum period (9), which may be related to malnutrition, alcohol use and smoking, suicidal behaviors, and postpartum depression, as well as risk factors for pre-eclampsia, premature labor, and low birth weight (2, 14, 15).

In addition, the changes that occur in women's bodies during pregnancy and postpartum can have consequences on their body image. Research suggests that body dissatisfaction can worsen at specific periods, such as early pregnancy (16, 17), in the middle (18, 19), at the end (20, 21), or in the postpartum period (3, 22). In these cases, pregnancy and postpartum can intensify or cause a negative relationship with the body (1, 3). Recently, authors reported on the importance of evaluating body dissatisfaction during pregnancy as a predictor of the onset of prenatal depression in Polish women (10). However, the authors pointed out that, in clinical practice, this risk factor should be considered in combination with positive maternal attitudes, not separately (10).

In this sense, previous study found body dissatisfaction in 45% of pregnant women with high BMI and high parity (23). Other studies have also evaluated the association of body image with other psychological variables in pregnant women who asserted that depression, low self-esteem, and higher BMI can trigger negative bodily attitudes in this population (24–26). In addition, current studies showed that more-troubled sociodemographic conditions can be considered as risk factors for negative mental health (9, 27). No family support, unplanned pregnancy, multiparous, single women, having contemplated abortion, low education, and suffering from obesity showed to be alarming conditions for low self-esteem, poor body image, and greater depressive symptoms (9, 27). The occurrence of these issues during this phase of a woman's life can be prolonged and compromise the health of the mother, fetus, and child.

Although body image includes both positive and negative characteristics, much emphasis has been given to the negative aspects of this construct (28). Positive body image can promote and increase engagement in health-related behaviors (29, 30). Furthermore, the authors suggest that a positive body image protects physical health and psychological well-being. Therefore, there is a need to support people in appreciating, respecting, celebrating, and honoring their bodies (31, 32) during all stages of life, including pregnancy and the postpartum period (30).

In women's holistic health care, the assessment of body image during pregnancy and postpartum is recommended so that pregnant women have available assistance while dealing with the typical changes of these periods (1, 17, 33). It is worth emphasizing that promoting positive body image can be a non-pharmacological strategy that offers protection factors against psychological pathologies in pregnancy and the postpartum period. Body image and self-esteem can be important targets for prevention and treatment programs for postpartum depression. In addition, the influence of demographic conditions on body image, depressive symptoms, and self-esteem of pregnant women and postpartum women was already discussed in the literature (9, 11, 20, 23–27). Given these aspects, the aim of this study was to analyze the differences in body appreciation, depressive symptoms, and self-esteem between women in the three gestational trimesters and postpartum period, controlling by some sociodemographic conditions.

## Methods

### Ethical Approval

The study was approved by the Ethics and Research Committee at the Federal University of Juiz de Fora (registration number 50474115.1.0000.5147). All participants gave informed written consent and were offered no financial compensation.

### Study Design

This investigation presents a cross-sectional delineation and was carried out in different the five regions of Brazil (North, Northeast, Central, Southeast, and South). Data were collected between October 17th 2016 and March 1st 2017.

### Participants

We recruited a total of 1,861 adult pregnant women regardless of their gestational period, or postpartum women until 6 months after delivery. Women who did not fill out any of the three questionnaires were excluded. Thus, we excluded 438 women that did not answer one or more questionnaires. After missing data exclusion, 1,423 women aged between 18 and 52 years old (*M* = 29.22 ± 5.72) took part in the study. Among them, 97 (6.8%) were in their first trimester (*M* = 9.23 ± 2.53 gestational weeks); 449 (31.6%) were in their second trimester (*M* = 19.96 ± 3.60 gestational weeks), 538 (37.8%) in their third trimester (*M* = 31.93 ± 3.91 gestational weeks), and 339 (23.8%) were postpartum women (*M* = 85.45 ± 60.88 days after delivery).

Table 1 presents descriptive analyses of sociodemographic variables for each group. For all four groups, most women were married and had a good relationship with the baby's father, had family support, had planned the pregnancy, and did not think about interrupting the pregnancy.

**Table 1:** Relative frequencies for sociodemographic variable for each group.

	**First trimester**	**Second trimester**	**Third trimester**	**Postpartum**
Nutritional status				
Low weight	10.3	16.3	11.7	0.6
Eutrophic	44.3	40.3	37.5	42.8
Overweight	28.9	26.1	31.6	36.6
Obesity	16.5	17.4	19.1	20.1
Schooling				
Elementary school	9.3	6.0	5.4	2.7
High school	40.2	38.6	35.9	31.9
Higher education	33.0	31.5	31.3	38.1
Post-graduate	17.5	23.9	27.4	27.3
Marital status				
Single	16.5	12.5	11.9	13.5
Married	83.5	86.4	86.8	85.0
Divorced/widowed	0	1.1	1.3	1.5
Parity				
Nulliparous	55.2	64.4	61.5	71.4
Multiparous	44.8	35.6	38.5	28.6
Relationship with the baby's father				
Bad	1.2	3.8	4.8	5.0
Good	98.8	96.2	95.2	95.0
Family support				
No	3.1	0.9	2.1	3.8
Yes	96.9	99.1	97.9	96.2
Gestation planning				
No	46.4	40.1	41.4	39.2
Yes	53.6	59.9	58.6	60.8
Initial desire to have an abortion				
No	90.7	93.8	95.7	93.5
Yes	9.3	6.2	4.3	6.5

### Procedures

The researchers contacted many professionals offering services to pregnant and postpartum women (obstetricians, event organizers, and health care institution owners). In obstetric clinics and health institutions, women answered the questionnaires while they were in the waiting room. In the case of events (courses, fairs), a trained team approached women individually in places reserved for rest or food. The participants took ~20 min to complete the questionnaires.

### Instruments

#### Demographic Questionnaire

Women provided demographic information such as age, education level, marital status, relationship with the baby's father, family support, gestation planning, and initial desire to have an abortion. Pregnant women also reported their gestational age in weeks and postpartum women provided the delivery date. Participants self-reported their weight and height data, which we used to calculate their Body Mass Index (BMI) and to classify these participants as low weight, eutrophic, overweight, and obese.

#### Body Appreciation Scale

We used the Body Appreciation Scale (BAS) (34) to assess body appreciation. The 13-item scale is a self-report instrument with 5 Likert response options. In our study, we found a high internal consistency (α = 0.903) for this measure (35, 36).

#### Rosenberg Self-Esteem Scale

We applied the Rosenberg Self-Esteem Scale (RSS) (37) to evaluate the participant's self-esteem. This scale is composed of 10 items that use a four-point Likert scale. The scale demonstrated good internal consistency (α = 0.863) when used in this study (35, 36).

#### Beck Depression Inventory

We used the Beck Depression Inventory (BDI) (38) to verify the presence of depressive symptoms through 21 items. BDI reliability was considered adequate (0.866) (35, 36).

### Statistical Analyses

We conducted this analysis in Statistical Package for the Social Sciences (SPSS) version 28.0, and in all cases the level of significance adopted was *p* < 0.05. Mean, standard deviation, median, minimum, and maximum were calculated for each variable in the study. Also, we analyzed absolute and relative frequencies for the category variables. We evaluated the scales' internal consistency through Cronbach's alpha. This was followed by the Kolmogorov Smirnov normality test, and inspection of asymmetry and kurtosis of the scores obtained. Based on previous literature, more troubled sociodemographic backgrounds can be more harmful to psychological vulnerability during the perinatal period (9, 11, 20, 23–27). Preliminary analysis was performed to test this hypothesis carrying out Mann–Whitney test and Kruskal–Wallis test in order to verify the existence of any difference between the groups for each outcome (BAS, RSS, and BDI). We run a non-parametric Quade test (ANCOVA), adjusting the effect of the variables that showed difference in the preliminary analysis on each outcome, between the four groups (first, second, third trimester, and postpartum). For all the comparative analyses, the size of the effect was determined by Cohen's *d* (39). We considered small, medium, and large effect sizes values lower than 0.5, between 0.5 and 0.79, or ≥0.8, respectively (39, 40).

## Results

### Preliminary Analysis

Table 2 shows the results regarding differences in the outcomes compared for nutritional status, educational level, marital status, parity, relationship with the baby's father, family support, gestational planning, and initial desire to have an abortion.

**Table 2:** Preliminary analysis of differences in the outcomes based on demographic variables.

	**BAS**	***p***	**RSS**	***p***	**BDI**	***p***
Nutritional status						
Low weight	54.90 ± 7.13	<0.001^*^	24.22 ± 5.02	<0.001^*^	7.08 ± 5.62	<0.001^*^
Eutrophic	52.77 ± 8.59		20.01 ± 9.06		8.25 ± 6.75	
Overweight	49.72 ± 9.79		19.43 ± 9.08		9.50 ± 7.54	
Obesity	46.36 ± 10.09		19.54 ± 8.19		10.51 ± 8.45	
Educational level						
Elementary school	51.66 ± 9.66	0.160	20.22 ± 6.38	0.163	10.50 ± 10.20	0.199
High school	51.71 ± 9.61		20.19 ± 8.10		9.01 ± 7.63	
Higher education	50.61 ± 9.57		19.98 ± 9.12		8.96 ± 7.10	
Post-graduate	50.49 ± 8.94		20.40 ± 9.34		8.51 ± 6.35	
Marital status						
Single	50.81 ± 10.50	0.340	20.16 ± 8.33	0.470	10.03 ± 9.19	0.730
Married	51.11 ± 9.29		20.17 ± 8.72		8.75 ± 6.97	
Divorced/widowed	47.87 ± 7.55		20.31 ± 9.27		10.06 ± 7.60	
Parity						
Nulliparous	51.54 ± 9.21	0.003^*^	19.81 ± 9.20	0.678	8.41 ± 6.37	0.038^*^
Multiparous	49.82 ± 9.84		20.23 ± 7.98		10.16 ± 8.85	
Relationship with the baby's father						
Bad	48.66 ± 11.29	0.188	18.84 ± 8.01	0.171	11.70 ± 10.19	0.085
Good	51.05 ± 9.43		19.91 ± 8.86		8.93 ± 7.18	
Family support						
No	49.61 ± 9.88	0.420	16.06 ± 9.59	0.022^*^	10.32 ± 10.97	0.899
Yes	51.08 ± 9.43		20.26 ± 8.65		8.89 ± 7.20	
Gestation planning						
No	49.62 ± 9.98	<0.001^*^	19.79 ± 7.94	0.002^*^	10.41 ± 8.17	<0.001^*^
Yes	52.02 ± 8.91		20.45 ± 9.14		7.91 ± 6.45	
Initial desire to have an abortion						
No	51.43 ± 9.18	<0.001^*^	20.32 ± 8.75	<0.001^*^	8.48 ± 6.84	<0.001^*^
Yes	44.26 ± 11.04		18.10 ± 6.89		16.26 ± 1031	

* *P < 0.05 is significant*.

### Covariance Analysis

Based on the preliminary analysis, we used as covariate in the main analysis only the variables that demonstrated an influence on the outcomes. Then, BMI, parity, gestational planning, and initial desire to have an abortion were used as covariates for body appreciation and depressive symptoms. BMI, family support, gestational planning, and initial desire to have an abortion were used as covariates for self-esteem.

We found significant statistical differences for body appreciation, depressive symptoms, and anxiety (Table 3). Women in their first trimester had lower body appreciation than women who were currently in their third trimester (*p* = 0.010). Pregnant women on their second trimester had lower body appreciation than those on third (*p* = 0.027). In addition, women during the postpartum period presented lower body appreciation when compared to those on their first (*p* = 0.045), second (*p* = 0.0001), and third (*p* = 0.0001) trimesters. Self-esteem was similar between groups of pregnancy trimesters, but it was significantly lower in the postpartum period group when compared to pregnant women in the different gestational trimesters (*p* = 0.012; *p* = 0.0001; *p* = 0.0001). Similarly, depressive symptoms did not vary through pregnant groups, but it was significantly higher in the postpartum period group (*p* = 0.036; *p* = 0.0001; *p* = 0.0001).

**Table 3:** Covariance analyses of body appreciation, depression symptoms, and self-esteem between the perinatal period adjusting covariates for each variable.

	**1st trimester**	**2nd trimester**	**3rd trimester**	**PP**	**F**	***p***	***d***		***p***
BAS	50.48 ± 10.56	51.96 ± 8.39	52.24 ± 9.01	48.09 ± 10.42	20.647	0.0001^*^	0.447	1st vs. 2nd	0.186
								1st vs. 3rd	0.010^*^
								1st vs. PP	0.045^*^
								2nd vs. 3rd	0.27^*^
								2nd vs. PP	0.0001^*^
								3rd vs. PP	0.0001^*^
RSS	23.54 ± 5.07	24.24 ± 4.68	24.09 ± 5.06	7.64 ± 5.73	14.205	0.0001^*^	3.245	1st vs. 2nd	0.266
								1st vs. 3rd	0.287
								1st vs. PP	0.012^*^
								2nd vs. 3rd	0.908
								2nd vs. PP	0.0001^*^
								3rd vs. PP	0.0001^*^
BDI	8.74 ± 6.54	7.97 ± 6.25	8.66 ± 7.67	10.70 ± 7.92	13.724	0.0001^*^	0.377	1st vs. 2nd	0.156
								1st vs. 3rd	0.223
								1st vs. PP	0.036^*^
								2nd vs. 3rd	0.700
								2nd vs. PP	0.0001^*^
								3rd vs. PP	0.0001^*^

*PP, postpartum; BAS, Body Appreciation Scale; RSS, Rosenberg Self-Esteem Scale; BDI, Beck Depression Inventory*.

* *P-value < 0.05 is significant*.

## Discussion

The goal of this study was to compare the differences in body appreciation, self-esteem, and depressive symptoms between women in the three gestational trimesters and in the postpartum period. The results showed significant differences for all the analyzed variables, demonstrating that the gestational period and the postpartum period have an impact on women's mental health. It is relevant to investigate body image from a positive perspective (29, 30, 32), seeking to prevent mental health disturbance in this population (41, 42).

Body appreciation was lower in pregnant women in their first trimester when compared to those on their third trimester. Furthermore, those on their second trimester had lower body appreciation than those on third. During the first gestational weeks, women may show less appreciation for their pregnant body as there is a feeling of weight gain without visibly appearing to be pregnant (2, 18). As the gestational trimesters progress, it is possible to accept and appreciate the increase in body dimensions. During these phases, mothers tend to prioritize their own health and baby's health, going beyond aesthetic concerns (1, 4). Pregnancy can be a reason for them to appreciate the functionality of their bodies, discouraging objectification (43). Furthermore, it seems that during pregnancy, the woman has a social allowance for the body to move away from the socially established standard of beauty, contributing to greater bodily acceptance (1, 2, 4).

However, our results showed lower body appreciation in the postpartum period group when compared to the three gestational trimesters groups, demonstrating that women had a less positive body image at this stage. Clark et al. (18) explained that there are no more excuses for larger body dimensions after the delivery. The fact that mothers feel fat can also be explained by the unrealistic expectations they have about the speed and ease with which the body will return to its pre-pregnancy shape (18, 44). In the same sense, previous studies found that mothers' body satisfaction decreased after delivery (25, 45). Thus, it is important not only to educate women about the expected weight and body changes in the postpartum period, but also to promote a positive body image, self-acceptance, and self-esteem after the delivery.

Findings for self-esteem and depressive symptoms were similar. These variables remained stable with no significant differences between the three gestational trimesters groups. Similarly, in a Brazilian longitudinal investigation, Meireles et al. (7) pointed out that self-esteem and depressive symptoms remained constant throughout pregnancy. Conversely, Chan et al. (25) found variations in the emotional state during pregnancy, with the third trimester showing more positive levels. Although the previous literature does not present a consensus regarding the variability of the pregnant woman's mental state, the fact is that the assessment of the mother's psychological aspects in the first trimester of pregnancy is extremely relevant since a troubled condition can persist throughout the pregnancy (11).

We also found a lower self-esteem and higher depressive symptoms in the postpartum group when compared to the gestational trimesters. These findings reinforce that this phase can be the most troublesome for the mental health of the mother. Previous studies have pointed out that the postpartum period involves low self-esteem and high depressive symptoms (9, 11, 46, 47). Furthermore, the theoretical model tested by Lee and Park (46) demonstrated that self-esteem and antepartum depressive symptoms are predictors of postpartum depression. Thus, if identified early, the woman can receive adequate support and avoid negative consequences in the postpartum period and during the baby's development (46, 48).

We must also acknowledge a few limitations of this study. The first limitation consisted in the collection of self-reported weight and height. It is possible that this information was under or overestimated by the participants. The anthropometric data measured are more reliable. However, researchers indicate the use of self-report measures to provide data on nutritional status in investigations with a large sample size (49). In addition, previous studies with pregnant and postpartum women also used self-reported anthropometric data (21, 42, 50). Second, the smaller sample size of first-trimester pregnant women is considered a limitation, considering the number of participants included in the other groups. It should be noted that there is greater difficulty in accessing this group of women as some of them discover the pregnancy late or even prefer not to expose the pregnancy. This is a common feature in comparative studies in this population (1, 17, 18). However, this study included 97 pregnant women in the first trimester. Finally, the cross-sectional design does not allow causal inferences and does not provide an assessment of the changes experienced by each of these women throughout pregnancy and the postpartum period. Despite these limitations, this study filled a gap in the literature because it examines positive body image, self-esteem, and depression symptoms of pregnant and postpartum women, which may contribute to the development and improvement of prevention strategies and gestational follow-up.

## Conclusion

We conclude that the postpartum period can be a difficult experience for women. After delivery, women showed low body acceptance, low self-esteem, and higher depressive symptoms compared to the gestational period. We recommend future studies that investigate the factors that can impact on body appreciation, self-esteem, and depressive symptoms, such as: race, ethnicity, breastfeeding during postpartum period, and type of delivery. We also indicate the need of longitudinal investigations that include the periods before, during, and after pregnancy.

Pregnancy and postpartum can be stressful events for women due to biopsychosocial changes. Multidisciplinary health teams play an important role in providing care to women. It is known that the obstetric examination is irreplaceable and extremely important for the physical health of the mother and baby. These teams should also be aware that some women may not cope with the rapid physical changes that are necessarily associated with pregnancy and the postpartum period, and that assistance at this time might be needed to promote a positive experience.

## Data Availability Statement

The raw data supporting the conclusions of this article will be made available by the authors, without undue reservation.

## Ethics Statement

The studies involving human participants were reviewed and approved by Ethics and Research Committee at the Federal University of Juiz de Fora. The patients/participants provided their written informed consent to participate in this study.

## Author Contributions

All authors listed have made a substantial, direct, and intellectual contribution to the work and approved it for publication.

## Funding

This research was funded by the Universidade Federal Rural do Rio de Janeiro.

## Conflict of Interest

The authors declare that the research was conducted in the absence of any commercial or financial relationships that could be construed as a potential conflict of interest.

## Publisher's Note

All claims expressed in this article are solely those of the authors and do not necessarily represent those of their affiliated organizations, or those of the publisher, the editors and the reviewers. Any product that may be evaluated in this article, or claim that may be made by its manufacturer, is not guaranteed or endorsed by the publisher.
